# Pentadecafluorooctanoic-acid-free polytetrafluoroethylene and mechanism of PFOA formation by γ-irradiation

**DOI:** 10.1038/s41598-020-70918-x

**Published:** 2020-08-18

**Authors:** Akihiro Oshima, Takayuki Tanaka, Hideki Nakaya, Ryosuke Senba, Kazuyuki Satoh

**Affiliations:** 1 Graduate School of Engineering, Osaka University, 2-1 Yamadaoka, Suita, Osaka 565-0871 Japan; 2 The Institute of Scientific and Industrial Research, Osaka University, 8-1 Mihogaoka, Ibaraki, Osaka 567-0047 Japan; 3 Process Technology Department, Chemical Division, Daikin Industries, Ltd, 1-1 Nishi-Hitotsuya, Settsu, Osaka 566-8585 Japan; 4 Technology and Innovation Center, Daikin Industries Ltd, 1-1 Nishi-Hitotsuya, Settsu, Osaka 566-8585 Japan

**Keywords:** Soft materials, Environmental chemistry

## Abstract

Low-molecular-weight (*M*_w_) polytetrafluoroethylene (PTFE) micropowder is added to wax for use in automotive equipment and printing machines and is produced by radiation-initiated degradation under atmospheric conditions. However, pentadecafluorooctanoic acid (PFOA) is produced as a by-product in concentrations greater than 25 ppb, which is problematic because PFOA does not degrade in the environment. Herein, we clarify the PFOA-formation mechanism and develop a manufacturing process for a novel low-*M*_w_ PTFE micropowder that does not contain PFOA (less than 5 ppb). The process uses combined irradiation and heat treatment in an oxygen-free atmosphere. Furthermore, PFOA-free PTFE micropowder can be produced on the 10-kg scale.

Pentadecafluorooctanoic acid (C_7_F_15_COOH, PFOA), its salts, and PFOA-related compounds have excellent surfactant properties and are used to give textiles water repellency and antifouling properties, as well as to prevent the scorching of kitchenware. However, they are not degraded in the natural environment and have high bioaccumulation properties. Thus, they are a cause of concern in relation to environmental pollution and human health damage. The Registration, Evaluation, Authorisation and Restriction of Chemical (REACH) legislation in the European Union (EU) will apply to the manufacture and sales of PFOA-containing products in the EU from July 4, 2020; hence it is necessary to reduce PFOA contamination to less than 25 ppb and that of PFOA-related compounds to less than 1,000 ppb^1^. Moreover, in the Stockholm Convention on Persistent Organic Pollutants (POPs), regulations concerning PFOSs are under consideration by the POPs Review Committee^2^ and will be added to Annex A at the ninth meeting of the Conference of the Parties to the Stockholm Convention (COP9)^3^.

Low-molecular-weight (low-*M*_*w*_) polytetrafluoroethylene (PTFE) micropowder is a powerful additive used in diverse industrial applications. With its high surface lubricity and anti-blocking properties, its micropowder is becoming accepted in industries that include food, textiles, pharmaceuticals, and electronics, and its global market was worth US$ 400 million in 2018^4^.

PTFE micropowder is produced industrially by the chain scission of its polymerised powder or moulded scraps induced by ^60^Co γ-rays, electron beam (EB) from accelerator or EB conversion X-rays irradiation in air. However, PFOA is produced at concentrations of more than 25 ppb as a by-product of radiation processing^5^. For this reason, PTFE micropowder does not comply with regulations and may cause serious problems in industrial applications. Therefore, to comply with regulations, a method for the manufacture of low-*M*_w_ PTFE with low PFOA contents (< 25 ppb) is required. Furthermore, its formation mechanism needs to be elucidated. In this paper, we clarify the formation mechanism and report a manufacturing process for a novel low-*M*_w_ PTFE with < 25 ppb PFOA. Note that ‘PFOA-free’ means below the detection limit (< 5 ppb) when analysed by liquid chromatography/mass spectrometry (LC–MS).

PTFE can be crosslinked by irradiation in its molten state in an oxygen-free atmosphere^6,7^, and the chemical structure of the obtained crosslinked PTFE does not contain oxygen according to Fourier-transform infrared (FT-IR) spectroscopy^8,9^, X-ray photoelectron spectroscopy (XPS),^9^ and ^19^F solid-state NMR^9,10^ analysis. Free radicals have not been trapped in the obtained crosslinked PTFE^11^. On the other hand, PTFE undergoes main chain scission when irradiated at room temperature under oxygen-free conditions^12–15^, and XPS^13^ has confirmed that the resultant PTFE contains oxygen, which is ascribable to atmospheric oxygen reacting with trapped alkyl and end-chain radicals^16^. Trapped radicals do not remain after irradiation in high temperature irradiation for crosslinking, while radicals exist in the sample in room temperature irradiation. That is, it is considered that trapped radicals remaining in PTFE are one of the causes for inducing PFOA. In particular, the scission efficiency is doubled when irradiated in air^15^, and low-*M*_w_ PTFE containing oxygen is produced. As a result, O_2_ is believed to induce the formation of PFOA (i.e*.*, > 25 ppb) during manufacture. Therefore, we turned our attention to solving this problem by examining the effect of irradiation and radical treatment in an oxygen-free environment.

## Results and discussion

Table 1 lists the amounts of PFOA emitted from the PTFE after γ-ray irradiation at 297 K in a vacuum. No clear dependence on PFOA concentration with absorbed dose was observed, and the PFOA is present at levels of less than or equal to 25 ppb. Furthermore, perfluoroalkyl carboxylic acids (PFCAs) group were detected in small amounts of 46 ppb in perfluorobutanoic acid (PFBA: C4), 26 ppb in perfluoropentanoic acid (PFPeA: C5), 20 ppb in perfluorohexanoic acid (PFHxA: C6), and 15 ppb in perfluoroheptanoic acid (PFHpA: C7), respectively. The amount of PFCAs above C9 (perfluorononanoic acid: PFNA) was below the detection limit. These are considered to be due to the effect of dissolved oxygen in matrix. For comparison, PTFE in opened glass ample was irradiated by γ-rays at a dose of 150 kGy in air; the amount of PFOA produced in this manner was 28.3 ppb, which exceeds the REACH regulation value. Moreover, PTFE enclosed in a polymer bag with air was irradiated with a dose of 150 kGy by γ-rays in air, and analysed for its PFCAs content. Large amount of PFCAs from C4 to C13 were detected. (see Supplementary Table S1 online). Furthermore, some commercial PTFE powders have been reported to contain in excess of 400 ppb PFOA^5^.

**Table 1:** Amount of emitted PFOA from PTFE after γ-ray irradiation at 297 K under vacuum.

Dose (kGy)	200	300	350	400	500
Emitted PFOA (ppb)	N.D.*	N.D.*	13.1	25.6	19.0

Irradiated samples were stored in vacuum at 297 K for 4 d.

*The detection limit under these experimental conditions is less than 5 ppb.

**Figure 1:**
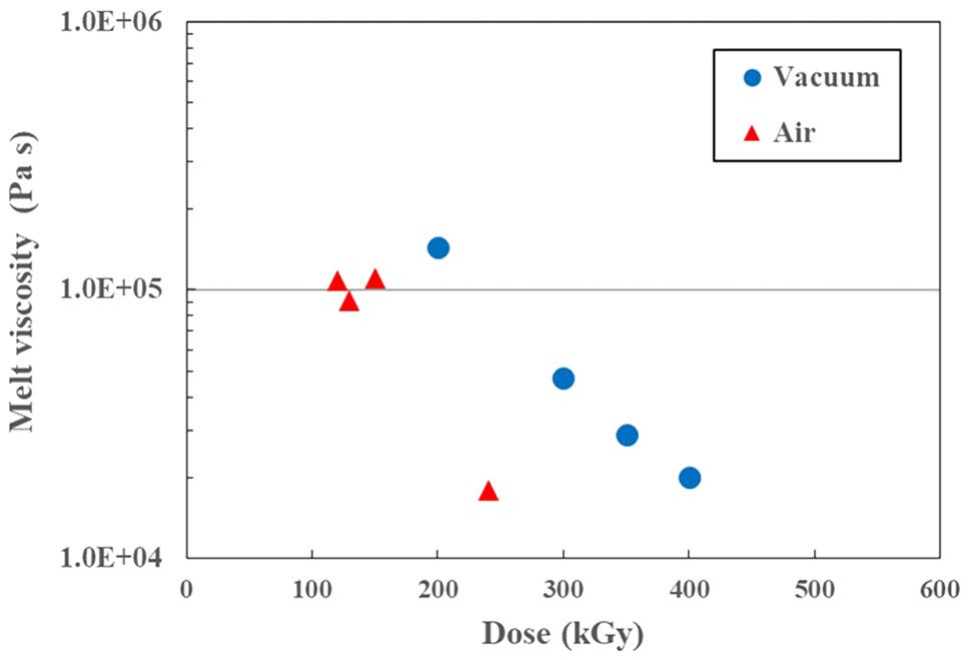
Melt viscosity of the PTFE micropowder as a function of dose after γ-ray irradiation at 297 K under vacuum and atmospheric conditions.

Figure 1 shows the melt viscosity of the obtained PTFE micropowder as a function of dose after γ-ray irradiation at 297 K under vacuum and atmospheric conditions. The melt viscosity values correspond to the molecular weight and are different for the vacuum irradiated and air irradiated samples. The lower melt viscosity means the lower molecular weight^17^. The melt viscosity of both vacuum and atmospheric conditions decrease with increasing absorbed dose, suggesting that the molecular weight decreases with irradiation. From the Suwa’s equation^18^ using differential scanning calorimetry (DSC) method, it is calculated that the molecular weight from the initial 1.73 × 10^6^ Da has become less than 5.2 × 10^5^ Da by 150-kGy γ-ray irradiation. The melt viscosity results indicate that the dose required under vacuum conditions is twice that required by irradiation in air. This effect of irradiation on melt viscosity is consistent with the results of tensile testing and thermal analysis using DSC reported previously^15^. The molecular weight is lower when irradiated in air because of increased scission by radiation-induced oxidation, and the obtained PTFE contains oxygen (e.g*.,* –CF_2_–COOH [1775 cm^-1^, 1813 cm^-1^, 3,557 cm^-1^] and –COF [1884 cm^-1^]), as revealed by FT-IR spectroscopy and reported previously^9,19^. Main chain scission is enhanced about twice by oxidation. That is, oxidative degradation does not occur on irradiation in an oxygen-free atmosphere.

The amount of PFOA produced by irradiation of PTFE under vacuum is significantly lower than that produced by irradiation in air. Thus, it is found that O_2_ is involved in the formation of PFOA during micropowder manufacture. However, even if PTFE is irradiated at 297 K under vacuum, it will still produce ± 25 ppb of PFOA. This is around the REACH regulation limit, and it is considered that the regulation value may not be able to be cleared under some conditions. One of the causes for this is thought to be the effect of dissolved oxygen in PTFE. Moreover, it is considered that the small amount of PFOA produced was induced by the oxidative reaction of the remaining trapped radicals in PTFE matrix after exposure to air. When the trapped radical in PTFE was stored in air and/or was carried out heat treatment in air, it is observed that PFOA was produced again. (see Supplementary Table S2 online) Therefore, the behaviour of the radicals trapped in the PTFE was evaluated using ESR spectroscopy.

**Figure 2:**
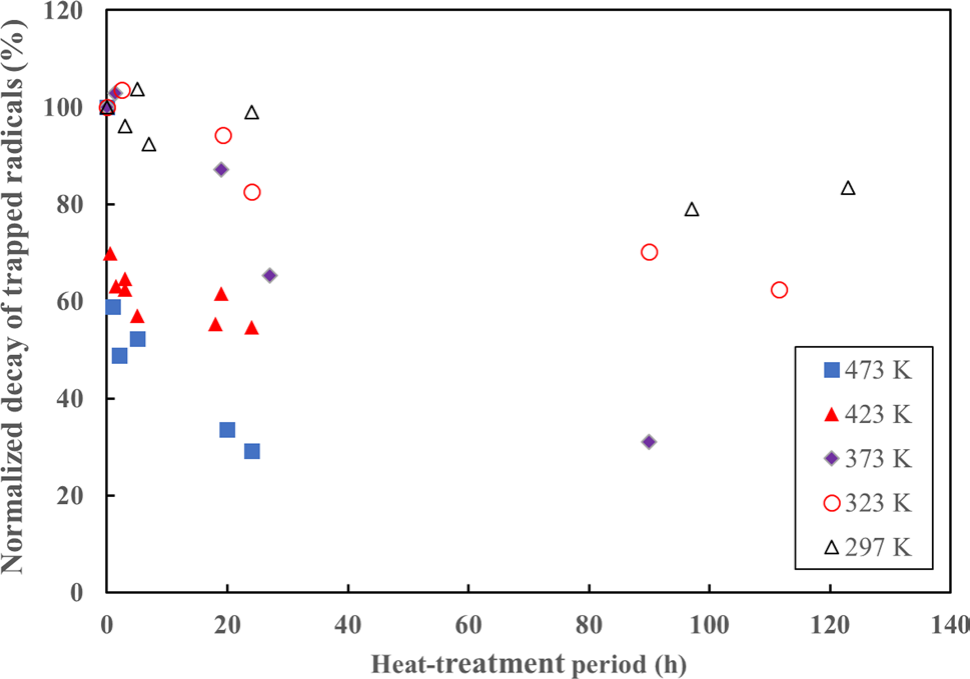
Normalised decay of trapped radicals in PTFE as a function of heat-treatment period after irradiation. γ-Ray irradiation was carried out with a 150-kGy dose under vacuum at 297 K.

Figure 2 shows the normalised decay of trapped radicals in PTFE as a function of the heat-treatment period after irradiation in a vacuum at 297 K. The initial radical yield was about 1.46 × 10^18^ spin g^-1^ with a dose of 150 kGy. The radical yield gradually decreased with time when heat treated at 297 K; in addition radical loss was observed to accelerate with increasing treatment temperature. The radical yield was significantly lower after heat treatment at 423 K, at 60% of the initial value after about 3 h.

Degrees of crystallinity over 90% were determined by DSC^20^ and X-ray diffractometry (XRD; scattering range *2θ* = 10—25° for crystallinity calculation with peak area method). In DSC/XRD analysis, since PTFE fine powder was originally highly crystalline, no significant difference before and after irradiation / heat treatment was observed in the DSC thermogram (heat of melting )/XRD spectrum (*2θ* = 18° and *2θ* = 40°). Radical annihilation proceeds even at temperatures below the melting point (600 K), which indicates that radicals are trapped not only in the crystalline phase but also in a para-crystalline phase or at interfaces and interchain regions of the crystallites.

Studies on the molecular motion of PTFE^21–23^ have shown that its transitions and relaxations are α (403 K), β (292 and 303 K), and γ (176 K); the γ relaxation is a glass transition, whereas the α relaxation is a transition in the molecular motions of interpolymer chains between crystallites, while β relaxation is a molecular conformational change in the crystallite. It is therefore a crystalline phase transition: β_1_ at 292 K is a first-order crystal phase transition in which the structure changes from triclinic to hexagonal because the molecular conformation changes from a periodicity of 13 to 15 –CF_2_– per 180° twists of the molecule. The β_2_ transition at 303 K is related to crystalline chain rotation.

From the viewpoint of molecular motion, the radical behaviour involves radicals trapped in crystalline regions slowly decaying above the crystal-phase transition temperature. Radical decay proceeds efficiency above the α relaxation temperature because of increased molecular motion, after which the trapped radicals remain in the low interaction area inside the crystals.

**Figure 3:**
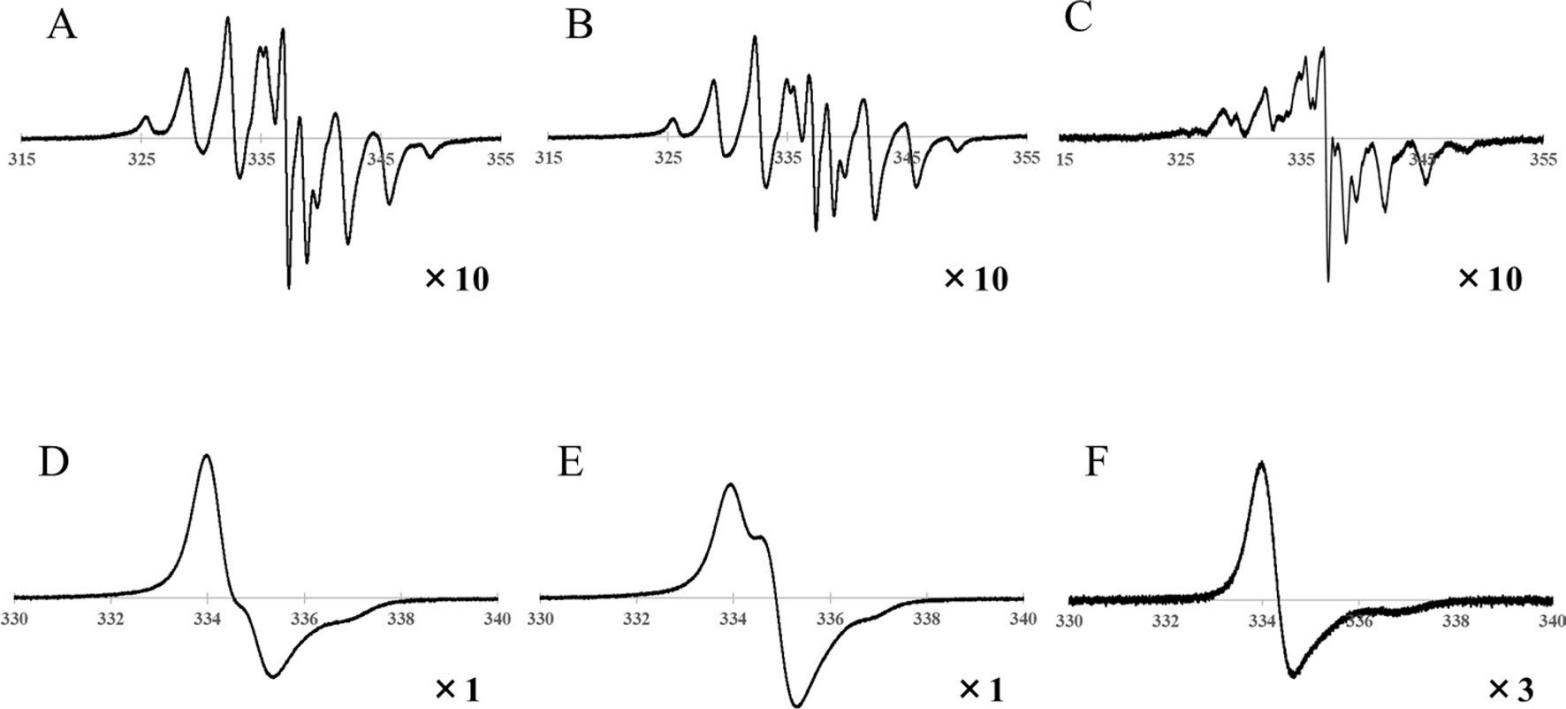
ESR spectra of PTFE samples after γ-ray irradiation with a dose of 150 kGy at 297 K. (**A**) PTFE 1 h after vacuum irradiation (radical yield: RY = 1.44 × 10^18^ spin g^-1^). (**B**) PTFE after vacuum irradiation and heat treatment under vacuum at 423 K for 24 h (RY = 7.83 × 10^17^ spin g^-1^). (**C**) Difference spectrum produced by subtracting [B] from [A]. (**D**) Exposed in air immediately after vacuum irradiation (RY = 1.06 × 10^18^ spin g^-1^). (**E**) Air irradiation. F: Exposed to air immediately after vacuum irradiation and then heat treated at 423 K in air for 3 h (RY = 1.53 × 10^17^ spin g^-1^).

Figure 3 shows the ESR spectra of various treated PTFE samples after 150-kGy γ-ray irradiation at 297 K. Figure 3A shows the ESR spectrum obtained 0.5 h after vacuum irradiation and is a superposition of a major double quintet signal and minor triple ones. The ratio of the double quintet to the triplet has been reported to be 10:1^24^. The double quintet and triplet are assigned to alkyl ($-{\text{CF}}_{2}-\dot{\text{C}}\mathrm{F}-{\text{CF}}_{2}-$) and chain-end ($-{\text{CF}}_{2}-\dot{\text{C}}{F}_{2}$) radicals, espectively^15^. The whole signal intensity decreases with increasing heat treatment time, as shown in Fig. 3B, and C shows the difference spectrum obtained by subtracting [B] from [A]. It is found that the triplet signal preferentially decreases compared to the double quintet signal; that is, the intensity of the triplet corresponding to chain-end radicals is preferentially lost with heat treatment under vacuum. The chain-end radicals are susceptible to molecular motion induced by the elevated temperatures, as mentioned above. As a result, the chain-end radicals are preferentially annihilated. In contrast, because the yield of alkyl radicals does not decrease significantly, even at 423 K above the α relaxation temperature, they must be trapped within the crystalline lattice, which would be expected to have lower molecular motion than the ends of the molecular chains. Of course, alkyl radicals trapped in the crystalline phase are also affected by molecular motion above the crystal phase transition temperature and will gradually decay with increasing heat treatment time.

As shown in Fig. 3D, the signals obtained for the sample exposed to air after vacuum irradiation are asymmetrical; they are superpositions of major asymmetric signals originating from alkyl radicals (chain-type peroxy radical, –CF_2_–CF(OO⋅)–CF_2_–) and minor symmetric radicals arising from chain-end radicals (scission-type peroxy radical, –CF_2_–CF_2_OO⋅)^15^. On the other hand, in the case of irradiation in air, the spectrum shows symmetrical signals corresponding to scission-type peroxy radicals^15^, as shown in Fig. 3E. These results indicate that scission-type peroxy radicals contribute to the formation of PFOA because vacuum irradiation significantly reduces the production of PFOA compared to irradiation in air. Because the chain-end radicals are preferentially removed by heat treatment under vacuum after vacuum irradiation, the production of PFOA can be reduced to below the detection limit by performing heat treatment above the α relaxation temperature following irradiation.

Studies of the radical formation and reactivity of PTFE^25^ have revealed that carbonyl-containing PTFE and alkyl radicals, including a minor components of chain-end radicals, are produced when peroxy radicals trapped in PTFE are heated beyond 353 K under oxygen-free conditions. Therefore, we suggest that the PFOA-induced scission-type peroxy radicals can be removed by heating under oxygen-free conditions, even if scission-type peroxy radicals are produced by exposure to the atmosphere after vacuum irradiation. On the other hand, heat treatment in air after vacuum irradiation reduces the radical yield, as was observed under vacuum conditions, and the spectrum remained asymmetric, as shown in Fig. 3F. That is, by heat treatment in air at 423 K, scission-type peroxy radicals with high molecular mobility induced from alkyl radicals and oxidative reactions produce carboxylic acid derivatives, including PFOA, and decay. The alkyl radicals trapped in the crystalline regions are re-oxidised and, as a result, cause the formation of chain-type peroxy radicals (see Fig. 3F). However, produced PFOA by heat treatment in air evaporates and is released into the atmosphere. As a result, PFOA cannot be detected in the obtained PTFE micropowder. The method of evaporating and removing PFOA by heat treatment can obtain PTFE without PFOA, but it is a clear violation of REACH regulation^1^.

Therefore, PTFE micropowder can be used in a high-temperature environment under atmospheric conditions. To control the emission of PFOA below REACH/POPs limits during commercial use, sufficient radical destruction by heat treatment under oxygen-free conditions or by capping treatment with a radical scavenger, such as hydrogen or halogen gas, or combined heating and capping treatments are required.

The amounts of PFOA emitted from PTFE and the melt viscosity after 400-kGy γ-ray irradiation at 297 K under vacuum followed by heat treatment at 297 and 423 K over 24 h are listed in Table 2. Heat treatment above the α relaxation temperature (403 K) does not affect the production of PFOA. Furthermore, the melt viscosity does not change significantly, and it is equivalent to those of conventional PTFE micropowder. Therefore, irradiation and heat treatment under oxygen-free conditions to remove trapped radicals in PTFE, in particular chain-end radicals, yields a PTFE micropowder that complies with REACH regulations. ESR analysis indicates that alkyl radicals are trapped even after heat treatment for 24 h, and chain-type peroxy radicals originating from alkyl radicals trapped in crystalline phases may induce scission-type peroxy radicals that form PFOA during prolonged storage. Moreover, in the case of high volume manufacturing (HVM) for commercial use, scission-type radicals that induce the production of PFOA may remain because of the influence of distribution of heat in the equipment and the like during heat treatment. Therefore, the removal of radicals, including scission radicals, by heat treatment or the use of a scavenger is crucial to reducing the amount of PFOA to non-detectable levels.

**Table 2:** Amount of emitted PFOA and melt viscosity. γ-Ray irradiation at 297 K with a dose of 400 kGy under vacuum.

Treated temperature (K)	Amount of PFOA (ppb)	Melt viscosity (Pa s)
297 K	25.6	29 × 10^3^
423 K	N.D.*	18 × 10^3^

Heat treatment was conducted at 297 and 423 K under vacuum over 24 h.

*The detection limit under these experimental condition is less than 5 ppb.

On the basis of the experimental results, we propose a PFOA-formation mechanism for each process. In the case of atmospheric irradiation (see Supplementary Fig. S1 online), main-chain scission of PTFE by radiation-induced oxidation occurs preferentially, as already reported^12,13,15^, and water in the air is directly ionised/excited by the radiation to produce H⋅/OH⋅^26,27^, which combine with *R*_*f*_*-*COF, and *R*_*f*_-COOH (including PFOA) to produce *R*_*f*_-CO-*R*_*f*_. Thus, PFOA is formed at levels that exceed environmental standards.

**Figure 4:**
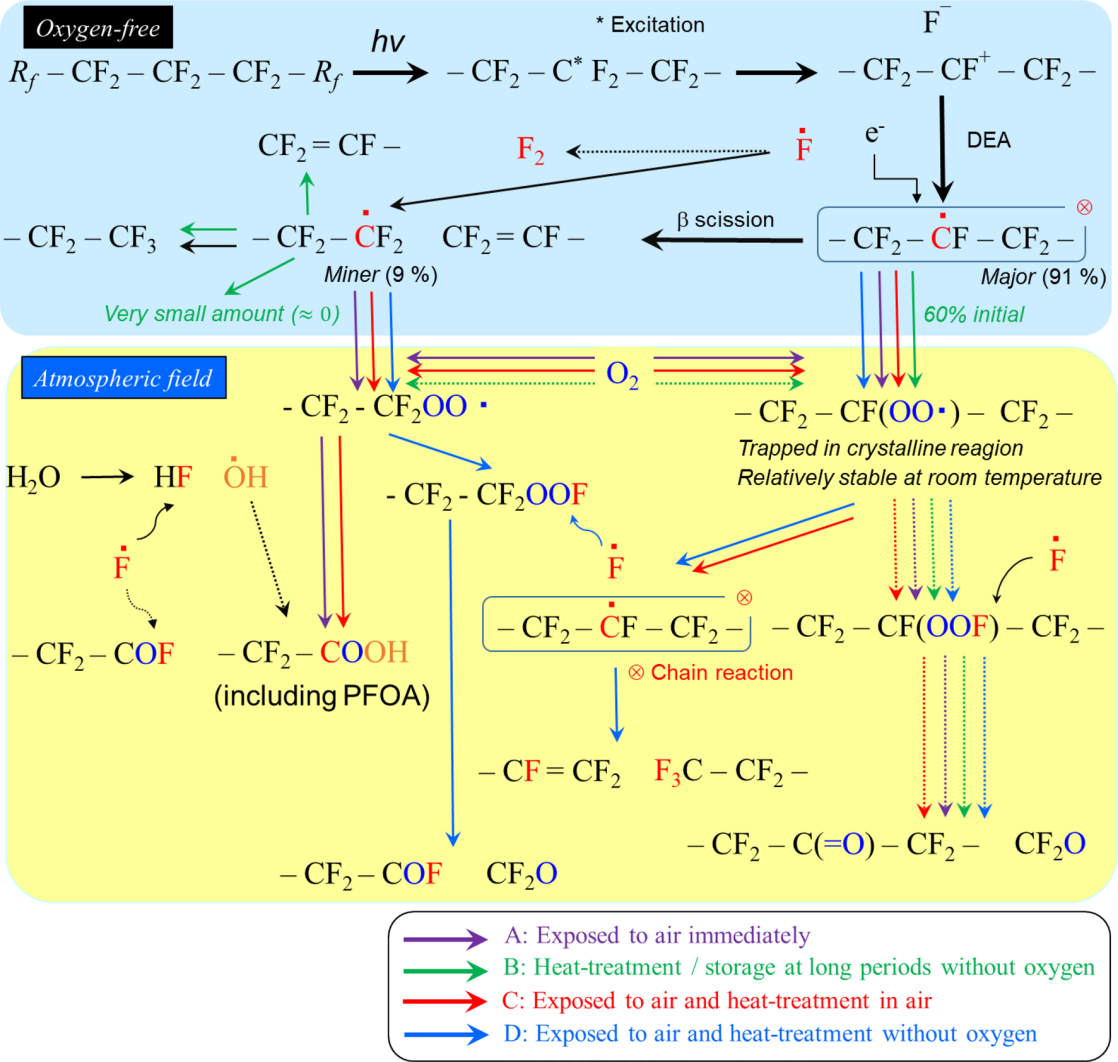
Reaction mechanism of PTFE irradiated in an oxygen-free atmosphere. Process A: Exposing to air immediately after irradiation. Process B: Heat treatment/storage for long periods in the absence of oxygen after irradiation. Process C: Exposure to air immediately after irradiation and then heat treated in air. Process D: Exposure to air immediately after irradiation and then heat treated without oxygen. Solid line: main reaction, Dotted line: minor reaction.

Figure 4 shows the presumed PFOA-formation mechanism for PTFE irradiation in an oxygen-free atmosphere. Irradiation under oxygen-free conditions results in the formation of alkyl radicals by dissociative electron attachment (DEA) because the fluorine electron acceptor easily accepts electrons and dissociates as F^-^ ions, which is the fastest and most important reaction of PTFE, as already reported^12,13,15^. Main-chain scission, which is induced by alkyl radicals, occurs via β-scission to produce *R*_*f*_*-*CF_3_, *R*_*f*_ -CF = CF_2_, and chain-end radicals.

In the case of air exposure immediately after irradiation (see Fig. 4 process A), the trapped alkyl/chain-end radicals are converted to their respective peroxy radicals at a ratio of 10:1^24^, which indicates that the remaining scission-type peroxy radicals react with water in the atmosphere with increasing time, producing around 25 ppb PFOA (*i.e.*, the REACH environmental limit.

After irradiation, during long-term storage or heat treatment under oxygen-free conditions (see Fig. 4 process B), the chain-end radicals preferentially decay, and the radical yield is reduced to about 60% or less of the initial level. Following exposure to the atmosphere, the scission-type peroxy radicals that result in the formation of PFOA, become a very minor component, and PFOA is formed at levels below the legal limit.

In contrast, when heat treatment is performed in the open atmosphere after irradiation (see Fig. 4 process C), the peroxy radicals react with oxygen and water in the atmosphere to induce thermal oxidative decomposition to generate scission-type peroxy radicals. As a result, the amount of PFOA produced exceeds the environmentally regulated limit.

After irradiation, when the sample is exposed to the atmosphere and then heat-treated without oxygen (see Fig. 4 process D), both types of peroxy radical are reconverted into alkyl radicals together with very small amounts of chain-end radicals, as reported previously^25^. In this case, because the radical yield is 60% or less of the initial value before exposure to air, the scission-type peroxy radicals, which are a PFOA-formation factor, are present as very minor components, and the amount of PFOA produced is much less than the REACH limit.

To achieve HVM based on the proposed PFOA-formation mechanism, process simplification and cost reduction are required. Irradiation and heat treatment in the absence of oxygen using glass or metal containers is costly and reduces HVM workability. Thus, the use of airtight polymer bags, such as polyamide or polyethylene terephthalate (PET), is appropriate to secure an oxygen-free environment. When the polymer material is irradiated by ionising radiation under oxygen-free conditions, hydrogen-based decomposition gases are emitted, as reported previously^28–31^. The use of a polymer bag would trap the hydrogen emitted upon irradiation under oxygen-free conditions, in turn quenching the trapped radicals in PTFE as a secondary effect.

As an example of this potential industrial process, 11 kg of PTFE was placed in tri-layer polymer bag comprising PET/aluminium/polyethylene and then evacuated. The sample was then irradiated with a 400-kGy dose of γ-rays at an oxygen concentration of 100 ppm or less, with subsequent heat treatment performed at 423 ± 5 K for 20 h in the same atmosphere. PFOA content in the obtained PTFE micropowder was less than 5 ppb, similar to the lab-scale experimental result mention above. Furthermore, PFOA content of this sample was less than 5 ppb even after a year stored in closed container under ambient condition.

## Methods

Homopolymerised PTFE fine powder was used in these experiments, and γ-ray irradiation was carried out at room temperature under vacuum and atmospheric conditions (see Supplementary Information for details). After irradiation, the induced trapped radicals were examined by electron spin resonance (ESR) spectroscopy, and the melt viscosity was evaluated with a capillary rheometer. Furthermore, the emitted PFOA from irradiated PTFE was evaluated by LC–MS (see Supplementary Information for details).

## Supplementary information

Supplementary information.
