# Supplementary material for: Pentadecafluorooctanoic-acid-free polytetrafluoroethylene and mechanism of PFOA formation by γ-irradiation
Source: Sci Rep. 2020 Aug 18;10:13940. doi: 10.1038/s41598-020-70918-x (PMC7435272; doi:10.1038/s41598-020-70918-x)
Supplement: Supplementary file 1 — Supplementary information. [file 41598_2020_70918_MOESM1_ESM.docx]

Supplementary Information

Pentadecafluorooctanoic-Acid-Free Polytetrafluoroethylene and Mechanism of PFOA Formation by γ-Irradiation

Akihiro Oshima,*^1,2^ Takayuki Tanaka,^1,3^ Hideki Nakaya,^1,3^ Ryosuke Senba,^3^ and Kazuyuki Satoh^1,4^

^1^Graduate School of Engineering, Osaka University, 2-1 Yamadaoka, Suita, Osaka, 565-0871, Japan

^2^The Institute of Scientific and Industrial Research, Osaka University, 8-1 Mihogaoka, Ibaraki, Osaka, 567-0047, Japan

^3^ Process Technology Department, Chemical Division, Daikin Industries, Ltd., 1-1 Nishi-Hitotsuya, Settsu Osaka, 566-8585, Japan

^4^Technology and Innovation Center, Daikin Industries Ltd., 1-1 Nishi-Hitotsuya, Settsu, Osaka, 566-8585, Japan

*Correspondence to: Akihiro Oshima, Graduate School of Engineering, Osaka University,

2-1 Yamadaoka, Suita, Osaka, 565-0871, Japan, e-mail: akoshima@sanken.osaka-u.ac.jp

**This file includes:**

**Results and Discussion**

Table S1.

Table S2.

Figure S1

Reference S1

**Methods**

Table S3.

**Results and Discussion**

**Supplementary Table S1.** Emitted various PFCAs after γ-irradiation with a dose of 150kGy in air.

| PFCAs | C4 | C5 | C6 | C7 | C8 | C9 | C10 | C11 | C12 | C13 | C14 |
| --- | --- | --- | --- | --- | --- | --- | --- | --- | --- | --- | --- |
|  | PFBA | PFPeA | PFHxA | PFHpA | PFOA | PFNA | PFDA | PFUnDA | PFDoDA | PFTrDA | PFTeDA |
| (ppb) | 196 | 201 | 480 | 277 | 590 | 265 | 165 | 97.2 | 71 | 13.9 | <5 |

**Supplementary Table S2**. Effect of re-heating of PTFE micropowder in air that cleared PFOA's REACH regulation value. The powder obtained by vacuum irradiation of 400kGy and radical treatment.

| Radical decay treatment | Re-heat treatment (K) | Amount of PFOA (ppb)* |
| --- | --- | --- |
| 297 K, 2days | - | 5 |
|  | 423 K, 18 h | 37 |
| 423 K, 18 h | - | < 5 |
|  | 423 K, 18 h | < 5 |

**
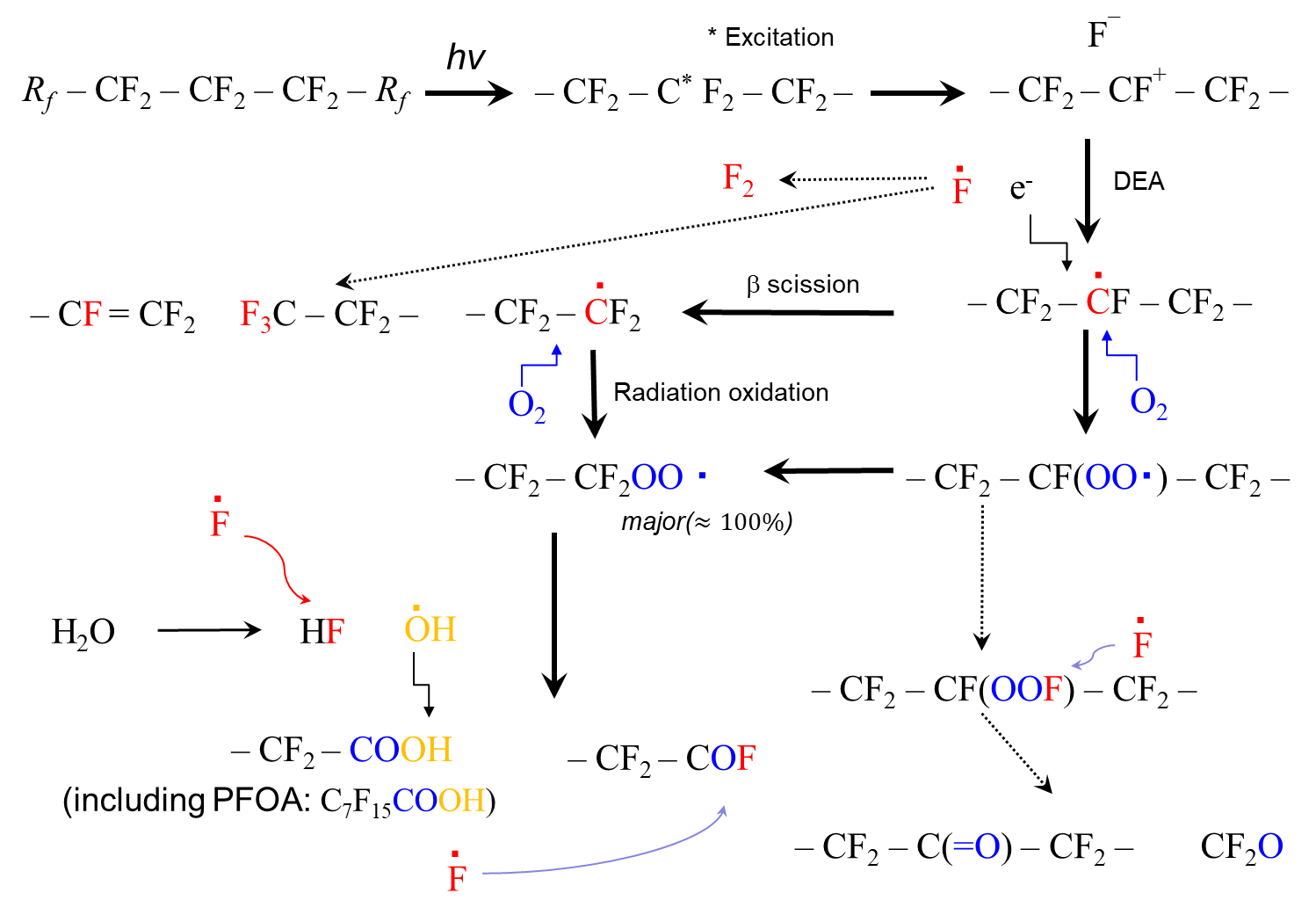
Fig. S1 |** Mechanism of PFOA formation after PTFE irradiation in air. Solid line: main reaction, Dotted line: minor reaction.

**Methods**

Materials

Commercially available homopolymerised PTFE powder (POLYFLON-PTFE) was supplied by Daikin Industries Ltd. The PTFE had a molecular weight of 1.73 × 10^6^ Da and an average particle size of ~500 µm. Molecular weight was determined from the heat of crystallisation of PTFE by DSC (TA-7000, Hitachi High-Tech Science).^S1^

γ-Ray irradiation

γ-Ray irradiation was carried out at ambient temperature (297 K) under vacuum and atmospheric conditions using a ^60^Co radioisotope source installed in cell No. 6 at the Takasaki Advanced Radiation Research Institute, Quantum Beam Science Research Directorate, National Institute for Quantum and Radiological Science and Technology (QST).

For the sample irradiated under vacuum conditions, the PTFE powder was sealed in a 5-mm-diameter quartz tube of for ESR spectroscopy (300.0 mg ± 10 mg) and a 18-mm-diameter glass ampoule for product analysis (10.0 g ± 1.0 g) after evacuation to less than 4.0 × 10^-3^ Pa for 24 h. The samples were irradiated to 500 kGy at a dose rate of 6.25 kGy h^-1^. For irradiation under atmospheric conditions, samples for both ESR and products analysis were placed in glass ampoules irradiated at the same dose rate.

Characterisation

*ESR spectroscopy.* Trapped free radicals were analysed using an X-band ESR spectrometer (JES-X330, JEOL) at 297 K. The ESR parameters, namely the microwave frequency, microwave power, sweep range of the magnetic field, field modulation width at 100 kHz, and time constant were set to 9.43 GHz, 0.04–0.1 mW, 337.0 ± 25 mT, 0.2 mT, and 0.1 s respectively. The total spin intensity was calculated by doubly integrating the spectrum and normalised using the instrument parameters. The radical yields (spin g^-1^) were determined from the spin intensity by calibration with TEMPO (2, 2, 6, 6-tetramethyl-1-piperidinyloxyl; 3.78 × 10^23^ spin g^-1^).

*Melt viscosity.* The melt viscosity of the PTFE micropowder was measured using a capillary rheometer CFT-500D (SHIMADZU EUROPA) at a pressure of 6.865 × 10^2^ kPa at (653 ± 1) K. The capillary 2 mm in diameter and 8 mm long. Viscosity was calculated from the pressure and transit time from standard position to a 5-mm extrusion point.

*LC-MS spectroscopy.* The amount of PFCAs extracted from irradiated PTFE was evaluated using ultra-high performance liquid chromatography (UPLC) (Acquity UPLC, Waters) coupled with negative ion electrospray mass spectrometry (Acquity TQ_D, Waters). PTFE extracts with masses of 1.0 ± 0.05 g were extracted by sonication for 5 min with 5 mL of acetonitrile (> 99.9% (GC), Kanto Chemical) in a 15-mL disposal vial tube. A 1.7-µm Acquity UPLC BEH C18 column (Waters) was used at 313 K . Upon injection of the 5 µL of sample, a gradient consisting of mobile phase A (2 mM acetonitrile) and mobile phase B (20 mM ammonium acetate aqueous solution) was initiated at a flow rate of 0.4 mL min^-1^ using the time program shown in Table S3. The parent-to-daughter-ion transition at 413 → 369 Da was measured for PFOA. Each sample was examined in duplicate.

**Supplementary Table S3.** UPLC gradient used for acetonitrile extractions at 313 K.

| Time (min) | A (%) | B (%) |
| --- | --- | --- |
| 0 | 40 | 60 |
| 2.0 | 40 | 60 |
| 2.9 | 80 | 20 |
| 3.0 | 40 | 60 |

**Reference**

S1. Suwa, T., Takehisa, M. & Machi, S. Melting and Crystallization Behavior of Poly( tetrafluoroethylene). New Method for Molecular Weight Measurement of Poly (tetrafluoroethylene) Using a Differential Scanning Calorimeter, *J. Appl. Polym. Sci.* 17, 3253–3257 (1973).
